# Mapping the Global Evidence for Children’s Palliative Care: 2020–2025

**DOI:** 10.3390/children13101337

**Published:** 2026-10-01

**Authors:** Callie Daniels-Howell, Lizzie Chambers, Julia Downing

**Affiliations:** 1 International Children’s Palliative Care Network, Bristol BS2 8PE, UK; programme.manager@icpcn.org (L.C.); julia.downing@icpcn.org (J.D.); 2 International Children’s Palliative Care Network, Bloemfontein 9301, South Africa

**Keywords:** children, paediatric, pediatric, palliative care, research, evidence, global

## Abstract

**Highlights:**

**What are the main findings?**

- Peer-reviewed children’s palliative care research grew substantially between 2020 and 2025, but nearly three-quarters (73.2%) of publications focused on the WHO Americas and European regions, with the United States alone the focus of about a third of all publications during the review period.
- Over 100 countries, mostly low- and middle-income, had no publication focused on their population and no contributing author across the six years.

**What are the implications of the main findings?**

- Narrowing the gap between where children’s palliative care is researched and where it is needed will require investment in locally led research capacity and equitable authorship, rather than growth in publication volume alone.
- These findings offer a clear evidence base for setting future research agendas, pointing to specific regions, countries, and clinical areas where new research and locally led studies would most strengthen the global children’s palliative care evidence base.

**Abstract:**

**Background:** The peer-reviewed evidence base for children’s palliative care (CPC) has grown considerably in recent years, yet the extent to which this literature reflects the global distribution of children requiring palliative care has not previously been established at a field level. This mapping review characterises the CPC evidence base published between 2020 and 2025, with particular attention to the geographic distribution of study focus and authorship. **Methods:** A mapping review of peer-reviewed CPC publications was conducted using Scopus. An adapted mapping review framework was applied to guide searching, screening, and data extraction. Publications were analysed by year, geographic region, first and last authorship, methodology, clinical focus, and thematic content. **Results:** A total of 2339 publications were mapped. Publication volume grew across the period, demonstrating an increasingly diverse evidence base for the field. However, both study focus and first and last authorship were heavily concentrated in high-income settings: the World Health Organization (WHO) Americas and European regions accounted for the majority of publications and authors. The African region was markedly underrepresented relative to its burden of need. Over one hundred countries had neither any publication focused on their population nor any first or last authors. Substantial disparities were also evident within regions, with regional totals frequently driven by a single country, and gaps in clinical and thematic coverage were more pronounced in underrepresented regions. Meaningful intra-regional disparities in publication volume and first and last authorship were observed across all WHO regions. **Conclusions:** This review documents both the progress and the persistent gaps in the global CPC evidence base. The distribution of research continues to diverge from the distribution of need. Realising a more inclusive and representative body of scholarship will require sustained investment in locally led research capacity, equitable collaboration structures, and research agendas responsive to the priorities of under-resourced settings and the diverse communities they serve.

## 1. Background

Children’s palliative care (CPC) is an approach to the care of children and young people with life-limiting and life-threatening conditions, encompassing physical, emotional, social, and spiritual support for the child and their family from the point of diagnosis through to death and bereavement [1]. An estimated 21 million children globally would benefit from palliative care, with 10.6 million experiencing serious health-related suffering, the majority of whom live in low- and middle-income countries where access to such care remains severely limited [2,3,4]. Estimates suggest that only 5–10% of children globally have access to palliative care [2,3,5,6].

Research has been identified as a key priority for the field and highlighted as a key pillar of the World Health Organization’s (WHO) Conceptual Model for assessing the development of palliative care [7]. Here, research rests on the foundations of community empowerment and policy and is the ground upon which the ‘conceptual house’ of education and training, use of essential medicines, and service development are built to support people with serious illness. With the awareness of its central role in developing services, research is included as an indicator of palliative care development in global atlases of palliative care need and service provision, as well as other seminal reports on the development of palliative care globally and future priorities for the field [7,8,9]. As Harding et al. argued in their 2024 report assessing the development of palliative care since the 2014 WHO Resolution, research is critical for developing contextually relevant and appropriate care, particularly for children in underrepresented regions [8]. Where palliative care for all children, everywhere, is the goal, research is critical to determine the most appropriate, effective, and efficient models and approaches to meet the vast needs of those in resource-limited contexts [8].

The evidence base for CPC has developed considerably over recent decades, reflecting both the maturation of the field as a distinct clinical and research discipline and increasing attention from funders, policymakers, and international health organisations [5,10]. However, much of this evidence has been generated from within high-income settings. There is a continued call for the development of evidence from low- and middle-income countries alongside broader efforts to ensure that CPC research is inclusive, equitable, and responsive to the needs and contexts of local communities [2,8,11,12]. Specific gaps noted are in relation to models of care, outcome measurement, the inclusion of children’s perspectives, and the validation of tools across diverse settings [6,11,13,14].

Systematic and scoping reviews have examined discrete aspects of CPC practice and provision, including symptom management, models of care delivery, and the experiences of families and caregivers [15,16,17,18]. However, a comprehensive, field-level picture of the published CPC evidence base has not previously been produced. Without such an overview, it is difficult to assess the extent to which the growing body of CPC literature reflects the global diversity of children who require this care, or to identify the structural patterns and gaps that should inform future research investment. This mapping review therefore aimed to provide a structured overview of the CPC research literature published between 2020 and 2025, to identify gaps that may inform future research agendas, funding priorities, and capacity-building efforts.

## 2. Materials and Methods

Peer-reviewed CPC publications from 2020 to 2025 were mapped, with the aim of providing a current and comprehensive picture of the field’s evidence base. Specifically, it examined the geographic distribution of publications and first/last authorship, the methodological profile of the literature, the clinical and thematic focus of published research, and the extent to which the evidence base reflects the global distribution of children with life-limiting conditions. The findings are intended to inform the efforts of researchers, funders, policymakers, and advocacy organisations working to strengthen CPC research capacity and practice globally, with particular attention paid to settings and populations currently underserved by the existing evidence base.

Mapping reviews are well suited to this purpose. Distinct from systematic reviews that seek to synthesise evidence on a defined question, mapping reviews aim to characterise the scope, distribution, and nature of a body of literature, providing a structured basis for identifying areas of evidential concentration and gaps [19,20]. In fields that are multidisciplinary, globally distributed, and at varying stages of development across different contexts, this form of evidence synthesis offers particular value for research planning, priority-setting, and advocacy.

### 2.1. Search Strategy

An initial literature review was undertaken of the abstract and citation database Scopus in June 2025, identifying papers from 2014 onwards (Table 1). The full Boolean operator search strategy can be found in Appendix B.

A total of 5903 entries were identified and so the scope was reduced to publications from 2020 onwards. This shift allowed us to catalogue the evidence in more detail and give a snapshot of the current state of the field. All articles published between 2014 and 2019 (n = 1407) were removed, resulting in 4496 articles published between 2020 and 2025. This list was then screened for duplicates (n = 44), resulting in 4452 articles.

All screening and data classification were conducted by the first author. The thematic and methodological classification framework was developed collaboratively by the authorship team prior to data extraction and was refined through discussion as needed during the review process. Cases where classification was unclear or where additional interpretation was required were discussed among the authors to reach agreement. Independent duplicate screening and classification were not performed.

### 2.2. Inclusion and Exclusion Criteria

Publications were screened for eligibility in accordance with the inclusion and exclusion criteria (Table 2). Thus, 2453 articles were removed, leaving 1999 publications.

Following completion of the primary search, a Scopus alert was established using the same search strategy and Boolean operators. Newly indexed records were screened and extracted on an ongoing basis until 31 January 2026 to capture publications from 2025 that may not have been available or indexed at the time of the original search. This resulted in an additional 340 articles, leaving a total of 2339 articles (Figure 1).

### 2.3. Data Extraction

Articles were added to Zotero reference manager (Corporation for Digital Scholarship, Fairfax, VA, USA) and then integrated into a Notion (Notion Labs Inc., San Francisco, CA, USA) database that captured title, authors, date of publication, journal, format, abstract, and Digital Object Identifier (DOI). Notion was used to conduct the review to facilitate ongoing engagement and broader public use of the mapped evidence on the ICPCN Global Research Hub. This allowed for dynamic tagging, analysis, and visualisation for direct use and engagement by the field, a key step to advancing the visibility, collaboration, and impact of CPC evidence globally.

Articles were identified by first and last author, country and WHO region of authors’ listed affiliation, listed affiliated institution, and role, where available. Language of publication, geographic focus of the study by country and WHO region, methods, and topic were also identified. This was determined through review of key words, title, abstract, and methodology, with additional screening of introduction, results, or conclusion where needed to determine eligibility and publication details. Individual country names followed the WHO naming conventions and WHO region distributions [21] and World Bank income classification [22]. Studies that had a global focus, such as systematic or scoping reviews, were identified as ‘Global’ and all six WHO regions. Multi-national and multi-regional studies were assigned all relevant country and regional classifications; therefore, individual publications could contribute to multiple country and regional totals.

Any study where children and young persons aged 0–18 years were directly engaged as study participants was identified with ‘children’s perspectives.’ This did not include studies where proxy participants, such as parents or clinicians, were discussing their understanding of children’s perceptions. Where clinicians were the population or the focus of the study, this was identified with ‘Clinicians’. Due to the high volume of literature specific to nurses and nursing, a separate ‘Nursing’ tag was also used. Clinical foci of publications were also included as thematic identifiers. Broader clinical categories were used, such as ‘Oncology’ and ‘Haematology’ rather than specific diseases to facilitate higher-level categorisation across a large body of publications. ‘Unspecified Life-limiting/Life-threatening Illness’ was used for all studies where children with any life-limiting illness were the topic or population but specific disease profiles were not named.

Methodological features were identified using high-level categories using the layered approach seen in Figure 2. Broader categories were applied in this review to demonstrate higher-level trends in methodological approaches in the field. Specific methodological details were only noted where the publication identified this; for example, publications were only identified as ‘Multi-centre’ if the publication specifically noted this. Given the volume of publications and the broader focus of the review, detailed assessment of individual studies for methodological features that were not explicitly articulated was not possible.

Topic identification was not meant to be exhaustive but intentionally abstracted to the largest groupings to be appropriate to the high-level nature of the review. These were formulated based on key words attributed to articles by authors. Key words were combined and refined to place articles into thematic groupings that would be appropriate for the broader mapping aims of this review (Figure 3). The original key words are retained in the database of publications for cross-checking, validation, and future detailed review by topic, region, or broader methodological category.

## 3. Results

From 2020 to 2025, there were 2339 peer-reviewed publications in 17 languages, with 91.2% (n = 2134) of publications in English (see Table A1 for all languages). Publication numbers were relatively steady between 2020 and 2024, with the lowest number (n = 299) in 2020 and highest (n = 382) in 2022. The number of publications increased substantially in 2025 to 569 publications (Figure 4).

### 3.1. Geographic Distribution

Across all years (2020–2025), 42.2% (n = 986) of publications focused on the WHO Americas (AMR) region, followed by the WHO Europe (EUR) region with 31.0% (n = 724) of publications. The fewest publications were from the WHO Southeast Asia (SEAR) and African (AFR) regions. The WHO EMR, SEAR and AFR regions combined made up just 7.2% of publications (Table 3).

For all publications across all years (2020–2025), the United States of America was the country of focus for the highest number of publications and comprised 33.3% (n = 799) of all publications. Publications focused globally, primarily literature reviews, comprised 10.5% (n = 252) of all publications. The United Kingdom (inclusive of England, Wales, Scotland, and Northern Ireland) was the next most represented individual country with 7.4% (n = 178) of publications (Table 4).

Of the 30 countries with the highest number of publications focused on their context, six countries (Australia, China, South Korea, Singapore, Japan, and Malaysia) in the WPR region are represented. Only one country from the EMR region (Iran), one from the SEAR region (India) and one from the AFRO region (Uganda) are represented. Of the 30 countries with the most articles focused on their context, only one is lower-middle income: India, at number 11, with 39 (1.6%) publications, and only one is a low-income country: Uganda, at number 29, with 12 (0.5%) publications.

### 3.2. Authorship

Across all publications for all years, there were 2854 unique first and last authors. The largest percentage were affiliated with institutions in the AMR region, making up 41.5% (n = 1191), with EMR, SEAR and AFR together contributing just 8.0% of all authors. Across all years, there were 1672 unique first authors, with 43.5% (n = 730) of these affiliated in the AMR region and 36.9% (n = 618) affiliated in the EUR region. There were 1413 unique last authors. Similarly, 41.0% (n = 583) were based in the AMR region and 38.7% (n = 550) in EUR (Table 3).

Of individual countries, the United States made up almost a third of all authors with 30.8% (n = 885). This was followed by the United Kingdom, though this decreased substantially to 9.0% (n = 258). One other country from the AMR region appeared in the list of ten countries with the highest number of authors, Brazil, making up 3.3% (n = 95) (Table 4).

### 3.3. Underrepresented Contexts

Between 2020 and 2025, there were 47 countries globally that published five or fewer publications. Of these, 34.0% (n = 16) were high-income countries, 17.0% (n = 8) upper-middle income, 42.6% (n = 20) lower-middle income and 6.4% (n = 3) low-income. There were 107 countries that did not have any publications focused on their populations or contexts. Of these, 31.8% (n = 34) were in the AFR region, 22.4% (n = 24) in the EUR region, 19.6% (n = 21) in the AMR region, 15.0% (n = 16) in WPR, 7.5% (n = 8) in EMR, and 3.7% (n = 4) in SEAR. Moreover, 18.7% (n = 20) were high-income countries, 32.7% (n = 35) upper-middle-income, 25.2% (n = 27) lower-middle-income and 19.6% (n = 21) low-income (3.7% [n = 4] had no income classification due to insufficient data). There were also 107 countries with no first authors and 109 countries with no last authors. All 107 countries with no first or last authors also had no publications focused on their specific context (Table 3). See Table A2 for full data on authorship distribution.

### 3.4. Methodological Distribution

Across all publications from 2020 to 2025, empirical studies (quantitative, qualitative, and mixed methods) made up 57.7% (n = 1343) of publications (Table 5). Quantitative studies made up the largest percentage of publications (27.7%, n= 669), followed by qualitative studies (23.5%, n = 561). Reviews and syntheses made up 20.8% (n = 495) and commentaries made up 20.0% (n = 477) of publications. Excluding publications with a global focus, publications that employed quantitative and mixed-method methodologies were predominately focused on the AMR and EUR regions. Only 0.9% (n = 6) of studies using quantitative methods were focused on the AFR region and only 0.7% of mixed-methods studies (n = 1) were focused on the SEAR region. Qualitative studies and reviews/syntheses were relatively more distributed across regions (Table 5).

### 3.5. Topic Distribution

Studies with a focus on ‘Clinicians’ constituted the largest topic group, making up 8.12% (n = 527) of publications (Table 5). This topic group includes studies focused on clinical care providers’ experience, opinions, perspectives, or needs in provision of, referral to, or relation to CPC. This was followed by studies focused on parents’ experience. Other key topic areas included end of life care, peri/neonatal palliative care, care models, bereavement, death and dying, decision-making, needs assessments and communication. Distributions across geographic regions followed patterns for global distribution of all publications. Notably, there were no publications on communication focused on the EMR and SEAR regions (see Table A3 for full data on thematic distribution). By clinical area, the largest number of publications was focused on oncology (41.8%, n = 447) and publications focused on unspecified life-limiting/life-threatening illness made up 27.9% (n = 298) of studies (Table 5).

## 4. Discussion

This mapping review provides a high-level overview of the peer-reviewed evidence base for CPC published between 2020 and 2025. The findings reflect a field that has developed in both volume and scope alongside persistent patterns of inequity in the geographic and institutional distribution of publications and authors. Understanding the current state of progress and the gaps that remain is important for directing future research efforts effectively.

### 4.1. A Growing but Unevenly Distributed Evidence Base

The steady rise in publication volume across the review period is consistent with growing international recognition of CPC as a distinct field of clinical and research enquiry [8,12]. It should be highlighted that publications from 2025 were identified through both the primary search and ongoing Scopus alerts, which may have contributed in part to the higher publication volume observed in 2025.

The literature published between 2020 and 2025 reflects a clinical focus extending beyond oncology to include non-malignant and unspecified life-limiting conditions. This is important given that most children requiring palliative care globally live with non-malignant conditions rather than cancer, and an evidence base that reflects this population can better support the development of care models suited to those most in need of them [2,13,23]. The corpus also includes substantial attention to care models, bereavement, and needs assessment, highlighting the multidimensional nature of contemporary CPC research and its relevance to holistic, family-centred care [1]. Methodologically, the literature draws on a mix of quantitative, qualitative, and mixed-methods traditions, alongside a substantial body of reviews and commentary. This diversity is an encouraging reflection of the multifaceted evidence needs of this interdisciplinary field.

### 4.2. Geographic Representation

Growth in publication volume alone does not ensure the depth or contextual breadth of evidence needed to inform practice across diverse settings. The most consistent finding of this review is the concentration of study focus in the WHO Americas and European regions. Within these, publications were focused on a small number of high-income countries, most notably the United States, which alone was the focus of roughly a third of all publications identified. This concentration intensified once reviews with a global scope were excluded, indicating that primary, context-specific research is even less evenly distributed than aggregate regional figures suggest. The pattern extended beyond overall publication counts to the breadth of countries represented: the African region, despite comprising the largest number of countries of any WHO region, had the smallest proportion of its countries represented by even a single publication. However, this number has increased since Harding et al.’s 2010 review, which found only five peer-reviewed publications across the continent, and all from South Africa and Uganda [6,14].

This representation may partly reflect the relatively longer histories and degrees of CPC service provision and development in the Americas and European regions [3,7,24]. However, the disparities in the focus of publications and representation within first and last authorship roles reflected in these results extend beyond patterns of CPC service development. This distribution may suggest that factors beyond service development alone, including well-documented disparities in research infrastructure, funding access, academic networks, and publication opportunities, also systematically advantage research and researchers in well-resourced settings and might shape the current evidence base [25,26].

These disparities are apparent within even the most represented thematic and clinical focus areas. For instance, no publications addressing cardiologic illness, the fourth largest clinical focus category overall, were focused on the AFR, EMR, or SEAR regions, and no publications addressing neurological illness, the third largest category, were focused on the AFR region. Similarly, no publications addressing communication, one of the review’s ten most common thematic areas of focus, were identified for either the EMR or SEAR regions. Across all regions, several subfields and populations remain substantially underrepresented in the literature, such as humanitarian settings, rare diseases, and children’s perspectives. Methodological diversity was also not evenly available across settings: quantitative and mixed-methods approaches were concentrated in the AMR, EUR, and WPR regions, where high-income countries were demonstrated to dominate the evidence base. This suggests that the focus and type, and not only the location, of available evidence differs systematically across the field.

The consequence is an evidence base whose geographic scope does not reflect the global distribution of children with life-limiting conditions, the majority of whom live in low- and middle-income countries [2,24]. Developing a more geographically and culturally diverse evidence base is critical if CPC is to be delivered in ways that are genuinely responsive to the values, beliefs, and meaning-making frameworks of children and families across different contexts [11].

### 4.3. Authorship Representation

A comparable, and in some respects more pronounced, concentration was evident in authorship, with most first and last authors affiliated with institutions in the same two regions that dominated study focus. This mirrors a well-documented pattern in global health research where researchers affiliated with institutions in low- and middle-income countries, and particularly in sub-Saharan Africa, remain underrepresented in senior authorship positions relative to their populations’ share of global disease burden [8,11,25,26]. Encouragingly, the review identifies first and last authors from a growing range of middle-income countries among the 30 highest most represented, including Brazil, India, Türkiye, China, and Iran, suggesting that the community of researchers contributing to the field’s evidence base is becoming more internationally varied. However, authors based in the AMR and EUR regions still accounted for 79.5% of unique first and last authors combined.

A further, distinct pattern was the substantial number of countries, concentrated in the African region, that had neither published output focused on their own population nor any author affiliated with an institution there across the entire study period. Given that most children needing palliative care live in low- and middle-income countries, this absence more plausibly reflects limited research capacity and infrastructure than an absence of clinical need. Efforts to map CPC service development globally have demonstrated that countries with no or limited CPC service provision and lack of CPC clinicians are less likely to have research [3,24]. This is a gap that several researchers have long identified as a principal barrier to reducing inequity in CPC provision globally [2,4,5,6,7,8,10,11,12].

When these authorship patterns are considered alongside the geographic focus of individual studies, increasing research attention to low- and middle-income settings has not yet been accompanied by commensurate growth in locally led scholarship across all regions, a gap that represents an important area for future development. Comparing the proportion of countries contributing at least one author with the proportion that were the focus of at least one publication is informative regarding the distinction between being studied and participating in research. In the SEAR region, 60.0% of countries were the focus of at least one publication, yet only 20.0% had any first or last author affiliated with an institution in that country—the largest gap between these two measures observed for any WHO region. By contrast, the AMR and EUR regions showed little difference between these measures.

These findings highlight the fact that geographic representation in the literature and participation in knowledge production are related but distinct dimensions of research equity. A country may be represented as a site of research without corresponding representation among those leading and shaping that research. Examining both dimensions provides a more nuanced understanding of the global CPC evidence base than publication volume alone. Scholarship drawing on decolonial research frameworks has argued that who leads, owns, and benefits from research is analytically and ethically distinct from where that research is conducted, and has called for a rebalancing of power dynamics such that research agendas are shaped by and with the communities where the need is greatest [25,26]. While evidence focused on a greater diversity of contexts is significant progress for the field, accompanying progress in the diversity of those leading that research is also an important area for the field’s future development.

This review only mapped first and last authors. As such, it indicates levels of representation in authorship positions commonly associated with leadership roles in publications but does not necessarily display all forms of research leadership, involvement or collaboration. Research capacity-building efforts may be evident through increased inclusion in publications as ‘middle’ authors that is commensurate with the time required to progress towards participation in first and last author roles. Reviewing these trends again in five years’ time will be critical to assess whether capacity-building efforts translate from involvement to ownership.

### 4.4. Intra-Regional Concentration

Regional-level figures obscure a further pattern of disparity within regions. The apparent dominance of the AMR region is attributable in large part to a single country: the United States accounted for 81.0% (799/986) of publications focused on the WHO Americas region, excluding those with a global focus. When combined with Canada and Brazil, these three countries accounted for 94.4% of all publications focused on the region, highlighting the substantial concentration of CPC research activity within a small number of countries. The remaining 5.6% of publications were distributed across the many other countries of North, Central, and South America and the Caribbean. A comparable pattern was observed in authorship, with the United States accounting for approximately two-thirds of first- and last-author instances attributed to the AMR region.

Comparable intra-regional concentration was evident in the regions with the smallest overall research output. Among the SEAR countries appearing in the top 30 countries of research focus, India alone accounted for approximately 75% of the region’s country-specific publications. In the AFR countries, Uganda, despite ranking twenty-ninth overall with 12 publications, accounted for approximately 27% of the region’s country-specific publications. While this represents meaningful growth in region-specific evidence, this is still a disproportionate share for one country relative to the size and population of the WHO African region.

Even within regions that appear comparatively well represented, evidence and authorship remains concentrated in a small number of countries. As demonstrated in the thematic, clinical, and methodological gaps observed, such assessments require nuanced analysis and response. This suggests that a research agenda oriented towards regional balance or global progress across thematic or clinical areas alone, without paying attention to which countries within each region are contributing to and benefiting from what research, may therefore be insufficient to address the underlying disparity [11,13].

### 4.5. Implications for Practice, Policy, and Research

As a mapping review, this study was designed to characterise the current evidence landscape rather than examine the causes of observed patterns. The implications discussed below therefore represent interpretations informed by the findings and existing literature.

A geographically representative evidence base is widely regarded as a precondition for the development, funding, and integration of children’s palliative care services into national health systems. The WHO’s framework for assessing palliative care development identifies research as one of the foundations supporting service development, education, and access to essential medicines [7]. Recent assessments of progress since the 2014 WHO resolution on strengthening palliative care have highlighted continued underdevelopment of research infrastructure in many regions as a barrier to progress [8].

This suggests that sustained growth in publication volume cannot on its own be assumed to narrow these disparities, given that the underlying geographic and institutional concentration described above remained broadly stable across the review period. Approaches described in the global health and palliative care literature may be relevant to addressing this gap. Authorship frameworks that make the contributions of in-country collaborators visible and require substantive local leadership on research conducted in low- and middle-income settings have been proposed as a mechanism for promoting more equitable knowledge production [26,27]. Reports from long-term, structured research partnerships indicate that directing funding and leadership roles to institutions in the country where research is conducted, alongside sustained investment in the broader research ecosystem, can shift patterns of research leadership over time [28]. Within palliative care specifically, strategies including regional centres of excellence, practice-based research networks, and structured South-to-South and North-to-South partnerships have been associated with growth in locally led research capacity in under-resourced settings [12,29].

Priority-setting exercises that directly involve researchers, clinicians, and communities from underrepresented regions may represent a further mechanism for ensuring that CPC research agendas reflect the priorities of the populations they are intended to serve—an approach consistent with earlier international consensus-building efforts in the field [13,23]. Considered alongside this literature, the patterns identified in this review suggest that closing the gap between the distribution of CPC research and the distribution of need will require sustained, deliberate investment in research capacity and leadership in underrepresented settings, rather than reliance on continued overall growth in publication volume alone.

### 4.6. Limitations

This review is subject to several limitations inherent to the mapping review methodology. Mapping the evidence across such a large time frame with limited resources holds limitations.

The findings are bounded by the search strategy and inclusion criteria applied; alternative scoping decisions would yield a different representation of the field. Although Scopus provides broad international coverage, this review was limited to publications indexed within this database and may not have captured relevant studies indexed elsewhere. The review is restricted to peer-reviewed publications, and therefore does not capture grey literature, including service audits or reports, policy documents, or conference proceedings, which may contain evidence more representative of practice in lower-resource settings. While the review included publications in any language, conducting the search itself in English alone may still have limited the representation of included studies. Additionally, publication counts function as an imperfect proxy for research activity, reflecting not only what is studied but the structural conditions that shape what is submitted and accepted for publication, conditions which are themselves subject to the inequities this review documents. Further, the geographic location of authors’ listed affiliations is not a fully representative proxy for authors’ geographic representation. Many authors hold multiple affiliations or may be affiliated in contexts outside of their country of origin, limiting the extent to which we can demonstrate research capacity within individual contexts using this metric.

Determining the appropriate scope of a mapping review in children’s palliative care involves substantive methodological judgement that reflects the nature of the field itself. CPC is genuinely multidisciplinary, drawing upon and contributing to evidence across a wide range of clinical and applied disciplines. A search strategy restricted to ‘children’s palliative care’ or ‘paediatric palliative care’ as key words risks excluding literature that is foundational to the field, including evidence on symptom management, family support, and advance care planning situated within related subspecialties. Conversely, an expansive inclusion strategy may conflate evidence specifically concerned with the provision of distinct CPC services with adjacent bodies of literature on supportive or psychosocial care embedded within other clinical contexts but not intended for CPC service provision specifically. The approach taken in this review sought to navigate these tensions, though the boundaries applied inevitably shape the resulting evidence map. Greater consensus on definitional and scoping conventions within the CPC research community would support more reliable cross-review comparisons and cumulative evidence synthesis over time.

This is not an exhaustive review and, as a high-level overview of the evidence, necessarily obfuscates some important details and distinctions within individual publications. For instance, the influence of the COVID-19 pandemic on the number and distribution of publications during the review period is unknown. This study maps the presence and representation of the published literature, but, as a mapping review, does not make any assessment of it. As a result, this review cannot fully speak to the quality or appropriateness of the literature, limiting the extent to which it can fully comment on the progress or state of the field.

## 5. Conclusions

The patterns identified in this review contribute to an ongoing and generative conversation within the CPC research community about how to ensure that its evidence base is as inclusive, equitable, and globally relevant as the care it seeks to support. This review set out to provide a comprehensive, field-level picture of the peer-reviewed CPC evidence base published between 2020 and 2025, examining not only what is being studied but by whom, from where, and using what methods. The findings demonstrate a substantial and diverse body of CPC literature, including research relating to neonatal palliative care, bereavement support, and life-limiting illness beyond oncology. Simultaneously, the review documents persistent and pronounced inequities in the geographic and institutional distribution of CPC scholarship and an evidence base that remains poorly aligned with the global distribution of children who require palliative care.

By identifying where research is concentrated, where it is absent, and where representation in first and last author roles in knowledge production remains uneven, this review provides a baseline against which future developments in the CPC evidence base can be assessed. This suggests that addressing these patterns is a collective responsibility for the CPC research community. Progress may require not only increased research activity in underrepresented regions but sustained investment in locally led research capacity, equitable collaboration structures, and a willingness to reorient research agendas towards the priorities of the communities most affected by gaps in CPC provision. The field has demonstrated that research originating in low- and middle-income settings can shape global practice; building the conditions for this to become the norm rather than the exception remains an important task ahead.

## Figures and Tables

**Figure 1:**
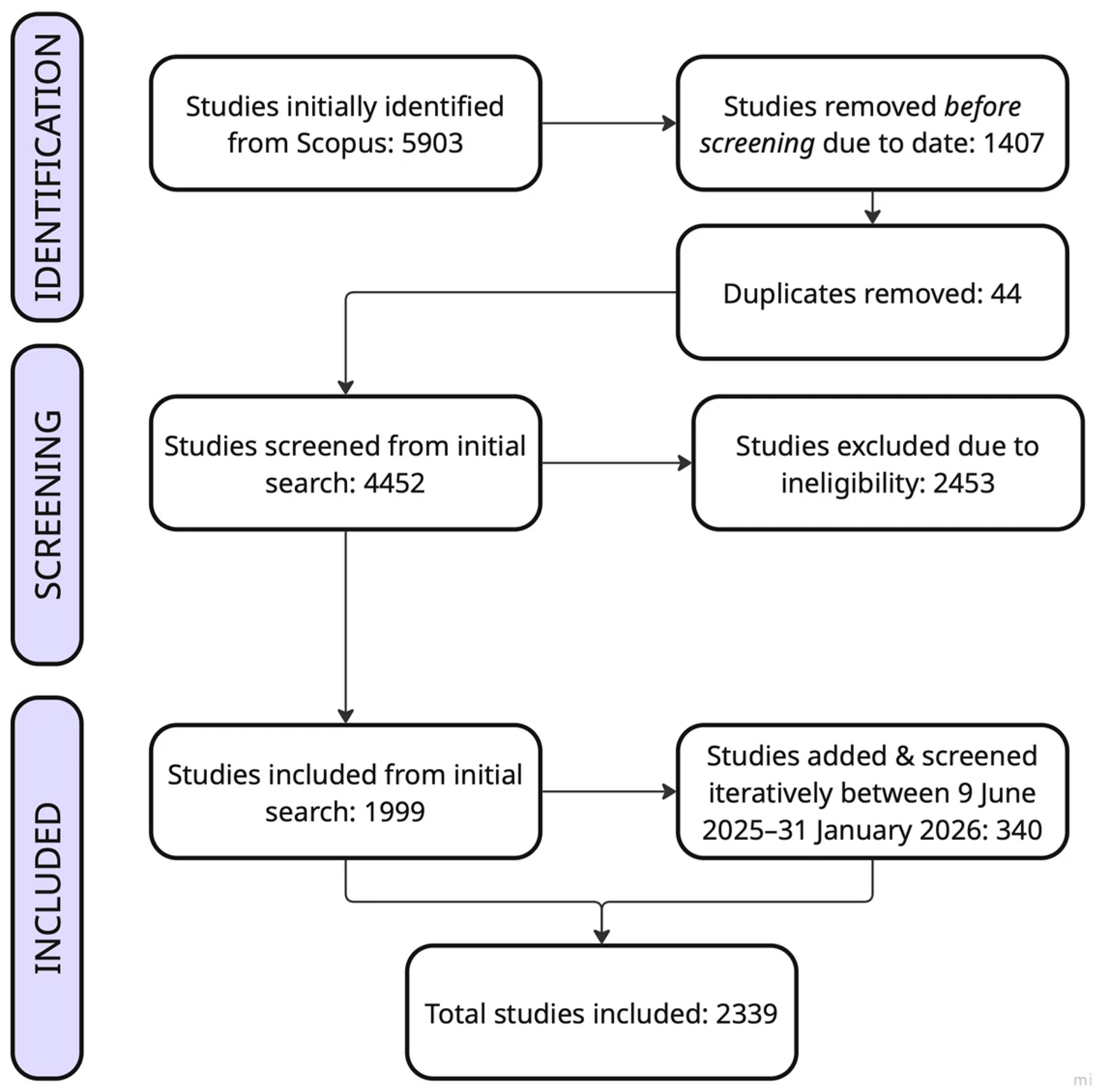
Mapping review flowchart.

**Figure 2:**
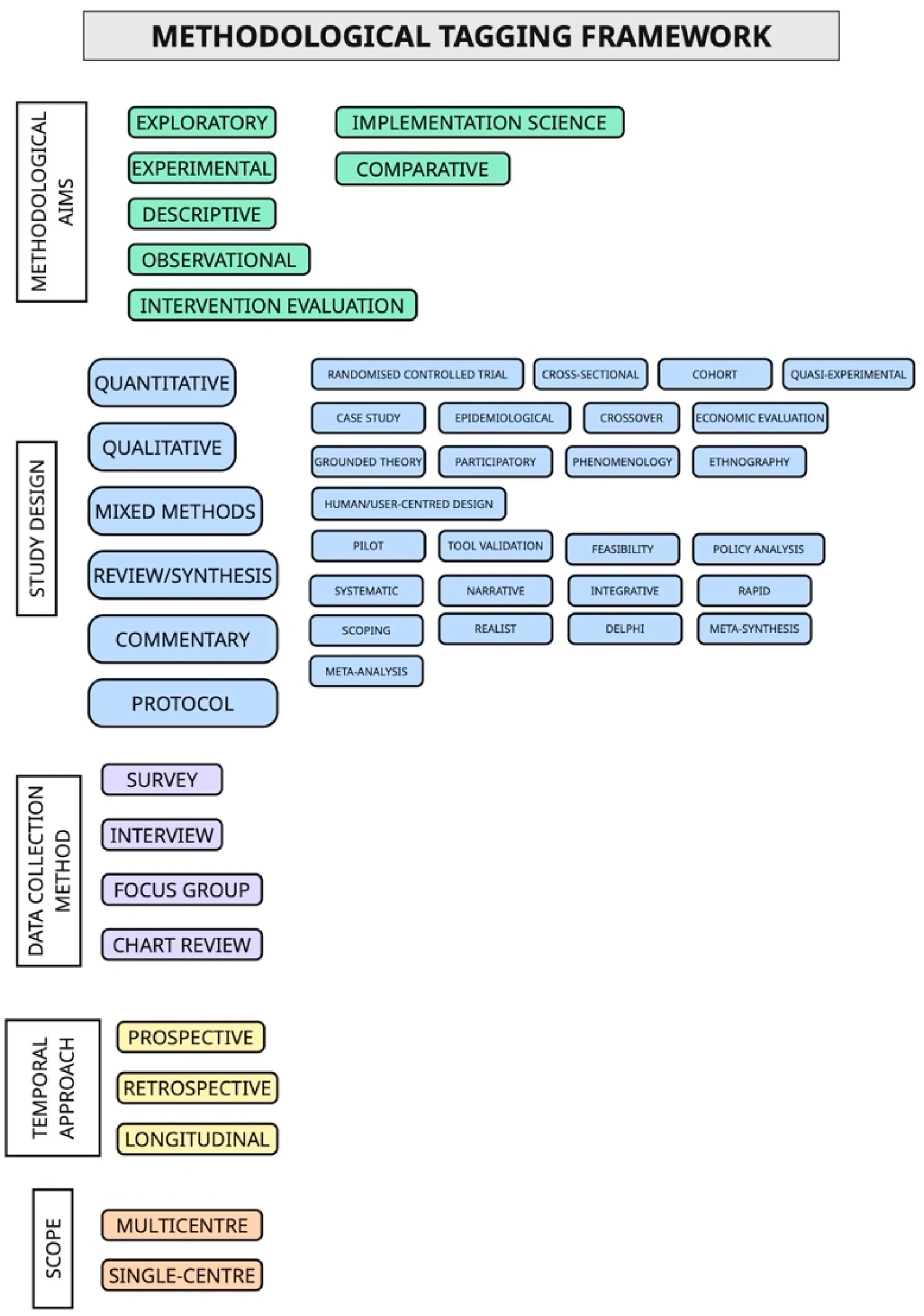
Methodological tagging framework.

**Figure 3:**
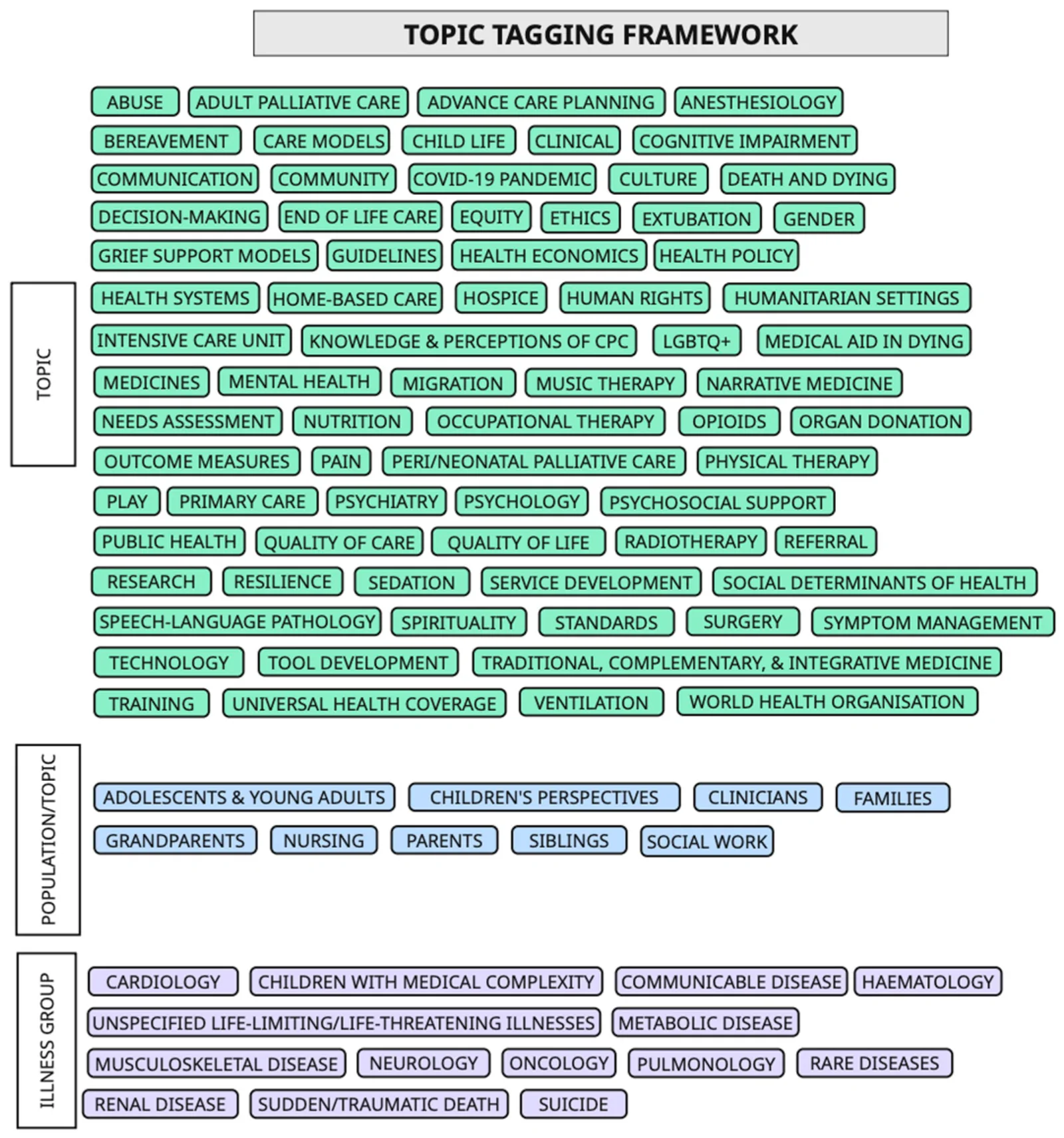
Topic tagging framework.

**Figure 4:**
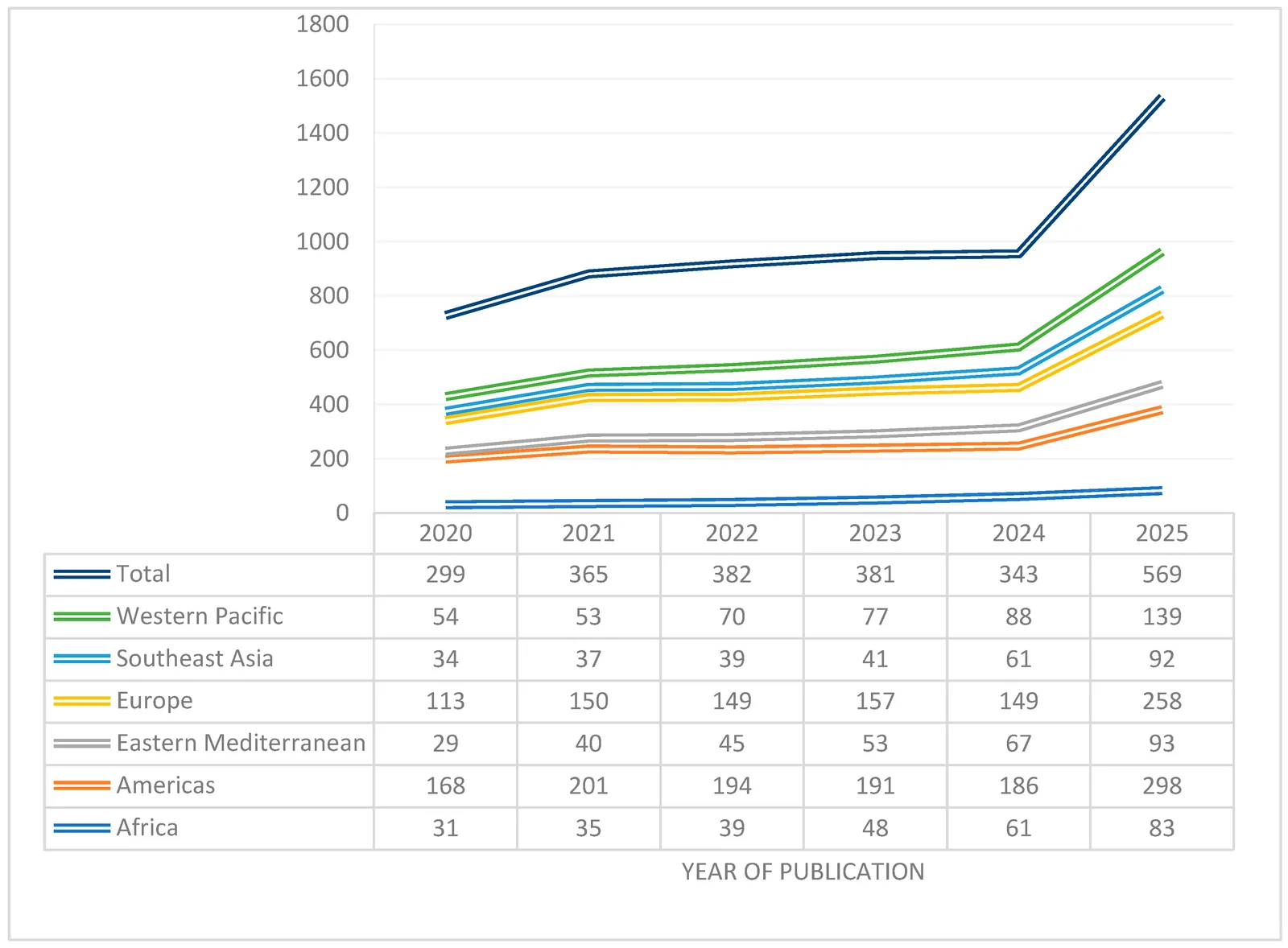
Publications by Year and WHO Region. Publications were assigned all relevant WHO regional classifications. Studies with multinational, multiregional, or global foci may therefore contribute to multiple regional totals. Consequently, the sum of regional counts exceeds the number of unique publications.

**Table 1:** Search term components.

Component	Terms
palliative care concepts	“palliative care”; “hospice care”; “end of life care”; “life-limiting condition*”; “supportive care”; “bereavement”; “grief”; “mourning”; “death of a child”; “child death”
population	child*; pediatric*; paediatric*; adolesc*; neonat*; parent*; family*; sibling*
publication year	>2014
exclusion terms	adult; geriatric; aged; elderly; dementia

* indicates a wildcard operator used to retrieve variations of a word stem. For example, child* retrieves child, children, and childhood, while paediatric* retrieves paediatric and paediatrics.

**Table 2:** Inclusion and exclusion criteria.

Criterion	Inclusion	Exclusion
**Topic and population**	Publications directly discussing CPC services, models, policies, needs, attitudes, or preferences among children and young people with life-limiting or life-threatening conditions and their families; publications in which CPC forms a component of broader paediatric care provision (e.g., CPC as a metric within a paediatric oncology needs assessment)	Publications not principally concerned with informing CPC; publications focused solely on adult populations; publications that only tangentially reference CPC without substantive discussion of or relevance to that care
**Related literature**	Publications that do not directly discuss CPC but inform components of its definition, where related to paediatric life-limiting or life-threatening illness (e.g., psychosocial support for children with medical complexity or their families)	Publications concerning supportive or psychosocial care not related to life-limiting or life-threatening illness in children
**Publication type**	Peer-reviewed journal articles	Grey literature, conference proceedings, book chapters, editorials, reports
**Date range**	2020–2025	Published before 2020 or after 2025
**Language**	Any	None

**Table 3:** Geographic distribution of publications and authors by WHO region.

	Total	Global (% of Total)	Africa (%)	Americas (%)	Eastern Mediterranean (%)	Europe (%)	Southeast Asia (%)	Western Pacific (%)
**Publications**								
**Total Publications**	n = 2339	n = 252 (10.8%)	n = 45 (1.9%)	n = 986 (42.2%)	n = 75 (3.1%)	n = 724 (31.0%)	n = 52 (2.2%)	n = 229 (10.0%)
**Countries with no publications**	n = 107		n = 34 (31.8%)	n = 21 (19.6%)	n = 8 (7.5%)	n = 24 (22.4%)	n = 4 (3.7%)	n = 16 (15.0%)
**Authors**								
**Total Authors**	n = 2854		n = 36 (1.3%)	n = 1191 (41.5%)	n = 120 (4.2%)	n = 1089 (38.0%)	n = 66 (2.3%)	n = 366 (12.8%)
**First Authors**	n = 1672		n = 21 (1.3%)	n = 730 (43.5%)	n = 65 (3.9%)	n = 618 (36.9%)	n = 36 (2.1%)	n = 207 (12.3%)
**Last Authors**	n = 1413		n = 16 (1.1%)	n = 583 (41.0%)	n = 59 (4.1%)	n = 550 (38.7%)	n = 34 (2.4%)	n = 180 (12.7%)

Publications were assigned all relevant country and WHO regional classifications. Studies with multinational or multiregional foci may therefore contribute to multiple country and regional totals.

**Table 4:** Highest number of publications and authors by country (top ten).

Country	Income	WHO Region	N = Total (%)
**Number of Publications Per Country**			
**United States of America**	High Income	AMR	n = 799 (33.3%)
**Global**	n/a	n/a	n = 252 (10.5%)
**United Kingdom**	High Income	EUR	n = 178 (7.4%)
**Italy**	High Income	EUR	n = 94 (3.9%)
**Australia**	High Income	WPR	n = 81 (3.4%)
**Canada**	High Income	AMR	n = 77 (3.2%)
**Spain**	High Income	EUR	n = 64 (2.7%)
**Brazil**	Upper-middle Income	AMR	n = 55 (2.3%)
**China**	Upper-middle Income	WPR	n = 53 (2.2%)
**Germany**	High Income	EUR	n = 53 (2.2%)
**France**	High Income	EUR	n = 41 (1.7%)
**Number of Authors Per Country (First and Last Combined)**			
**United States of America**	High Income	AMR	n = 885 (30.8%)
**United Kingdom**	High Income	EUR	n = 258 (9.0%)
**Canada**	High Income	AMR	n = 142 (4.9%)
**Australia**	High Income	WPR	n = 128 (4.4%)
**Spain**	High Income	EUR	n = 109 (3.8%)
**Italy**	High Income	EUR	n = 102 (3.5)
**Brazil**	Upper-middle Income	AMR	n = 95 (3.3%)
**China**	Upper-middle Income	WPR	n = 90 (3.1%)
**Germany**	High Income	EUR	n = 79 (2.7%)
**France**	High Income	EUR	n = 69 (2.4%)

**Table 5:** Publications by methodology and thematic focus areas across all publications.

	*Total (%)*
**Methodologies**	
*Quantitative*	n = 669 (27.7%)
*Qualitative*	n = 561 (23.5%)
*Review/Synthesis*	n = 495 (20.8%)
*Commentary*	n = 477 (20.0%)
*Mixed-Methods*	n = 154 (6.5%)
*Protocol*	n = 37 (1.6%)
**Thematic Areas**	
*Clinicians*	n = 527 (8.12%)
*Parents*	n = 509 (7.8%)
*End of life care*	n = 417 (6.4%)
*Peri-Neonatal Palliative Care*	n = 371 (5.7%)
*Care Models*	n = 285 (4.4%)
*Bereavement*	n = 252 (3.9%)
*Death and dying*	n = 190 (2.9%)
*Decision-Making*	n = 180 (2.8%)
*Needs Assessment*	n = 171 (2.6%)
*Communication*	n = 171 (2.6%)
**Clinical Focus**	
*Oncology*	n = 447 (41.8%)
*Unspec. Life-limiting/Life-threatening Illness*	n = 298 (27.9%)
*Neurology*	n = 102 (9.5%)
*Cardiology*	n = 55 (5.1%)
*Rare Diseases*	n = 49 (4.6%)

## Data Availability

The original contributions presented in this study are included in the article. Further enquiries can be directed to the corresponding author.

## Abbreviations

The following abbreviations are used in this manuscript:

CPC	Children’s Palliative Care
WHO	World Health Organization
AFR	(WHO) Africa Region
AMR	(WHO) Americas Region
EMR	(WHO) Eastern Mediterranean Region
EUR	(WHO) European Region
SEAR	(WHO) Southeast Asia Region
WPR	(WHO) Western Pacific Region

## Appendix A. Supplemental Data

**Table A1:** Full language data.

Languages	Total (%)	Global (% of Total)	Africa (%)	Americas (%)	Eastern Mediterranean (%)	Europe (%)	Southeast Asia (%)	Western Pacific (%)
**English**	n = 2134 (91.2%)	n = 244 (11.4%)	n = 43 (2.0%)	n = 936 (44.0%)	n = 73 (3.4%)	n = 600 (28.1%)	n = 52 (2.4%)	n = 209 (9.6%)
**Spanish**	n = 45 (1.9%)	n = 1 (2.2%)	n = 0 (0.0%)	n = 18 (40.0%)	n = 0 (0.0%)	n = 26 (58.1%)	n = 0 (0.0%)	n = 0 (0.0%)
**French**	n = 38 (1.6%)	n = 1 (2.6%)	n = 2 (5.3%)	n = 6 (15.8%)	n = 1 (2.6%)	n = 29 (76.3%)	n = 0 (0.0%)	n = 0 (0.0%)
**Portuguese**	n = 32 (1.4%)	n = 3 (9.4%)	n = 0 (0.0%)	n = 26 (81.3%)	n = 0 (0.0%)	n = 3 (9.4%)	n = 0 (0.0%)	n = 0 (0.0%)
**Italian**	n = 21 (0.9%)	n = 0 (0.0%)	n = 0 (0.0%)	n = 0 (0.0%)	n = 0 (0.0%)	n = 21 (100.0%)	n = 0 (0.0%)	n = 0 (0.0%)
**Mandarin**	n = 21 (0.9%)	n = 2 (9.5%)	n = 0 (0.0%)	n = 0 (0.0%)	n = 0 (0.0%)	n = 0 (0.0%)	n = 0 (0.0%)	n = 19 (90.5%)
**Russian**	n = 14 (0.6%)	n = 0 (0.0%)	n = 0 (0.0%)	n = 0 (0.0%)	n = 1 (7.0%)	n = 13 (93.0%)	n = 0 (0.0%)	n = 0 (0.0%)
**Czech**	n = 12 (0.5%)	n = 0 (0.0%)	n = 0 (0.0%)	n = 0 (0.0%)	n = 0 (0.0%)	n = 12 (100.0%)	n = 0 (0.0%)	n = 0 (0.0%)
**German**	n = 11 (0.5%)	n = 0 (0.0%)	n = 0 (0.0%)	n = 0 (0.0%)	n = 0 (0.0%)	n = 11 (100.0%)	n = 0 (0.0%)	n = 0 (0.0%)
**Swedish**	n = 3 (0.1%)	n = 0 (0.0%)	n = 0 (0.0%)	n = 0 (0.0%)	n = 0 (0.0%)	n = 3 (100.0%)	n = 0 (0.0%)	n = 0 (0.0%)
**Ukrainian**	n = 2 (0.1%)	n = 1 (50.0%)	n = 0 (0.0%)	n = 0 (0.0%)	n = 0 (0.0%)	n = 1 (50.0%)	n = 0 (0.0%)	n = 0 (0.0%)
**Danish**	n = 1 (0.0%)	n = 0 (0.0%)	n = 0 (0.0%)	n = 0 (0.0%)	n = 0 (0.0%)	n = 1 (100.0%)	n = 0 (0.0%)	n = 0 (0.0%)
**Dutch**	n = 1 (0.0%)	n = 0 (0.0%)	n = 0 (0.0%)	n = 0 (0.0%)	n = 0 (0.0%)	n = 1 (100.0%)	n = 0 (0.0%)	n = 0 (0.0%)
**Hungarian**	n = 1 (0.0%)	n = 0 (0.0%)	n = 0 (0.0%)	n = 0 (0.0%)	n = 0 (0.0%)	n = 1 (100.0%)	n = 0 (0.0%)	n = 0 (0.0%)
**Japanese**	n = 1 (0.0%)	n = 0 (0.0%)	n = 0 (0.0%)	n = 0 (0.0%)	n = 0 (0.0%)	n = 0 (0.0%)	n = 0 (0.0%)	n = 1 (100.0%)
**Norwegian**	n = 1 (0.0%)	n = 0 (0.0%)	n = 0 (0.0%)	n = 0 (0.0%)	n = 0 (0.0%)	n = 1 (100.0%)	n = 0 (0.0%)	n = 0 (0.0%)
**Turkish**	n = 1 (0.0%)	n = 0 (0.0%)	n = 0 (0.0%)	n = 0 (0.0%)	n = 0 (0.0%)	n = 1 (100.0%)	n = 0 (0.0%)	n = 0 (0.0%)

**Table A2:** Full publications and authorship data.

	Total	Global (% of Total)	Africa (%)	Americas (%)	Eastern Mediterranean (%)	Europe (%)	Southeast Asia (%)	Western Pacific (%)
**Publications**								
**Total Publications**	n = 2339	n = 252 (10.8%)	n = 45 (1.9%)	n = 986 (42.2%)	n = 75 (3.1%)	n = 724 (31.0%)	n = 52 (2.2%)	n = 229 (10.0%)
**Proportion of Countries with Publications**	n = 94/201 (46.8%)		n = 13/47 (27.7%)	n = 16/36 (44.4%)	n = 14/22 (63.6%)	n = 30/55 (56.4%)	n = 6/10 (60.0%)	n = 15/31 (48.4%)
**Countries with 5 or fewer publications**	n = 47		n = 9 (18.8%)	n = 8 (16.7%)	n = 10 (20.8%)	n = 10 (20.8%)	n = 5 (10.4%)	n = 6 (12.5%)
**Countries with no publications**	n = 105		n = 34 (31.8%)	n = 21 (19.6%)	n = 8 (7.5%)	n = 24 (22.4%)	n = 4 (3.7%)	n = 16 (15.0%)
**Authors**								
**Total Authors**	n = 2854		n = 36 (1.3%)	n = 1191 (41.5%)	n = 120 (4.2%)	n = 1089 (38.0%)	n = 66 (2.3%)	n = 366 (12.8%)
**First Authors**	n = 1672		n = 21 (1.3%)	n = 730 (43.5%)	n = 65 (3.9%)	n = 618 (36.9%)	n = 36 (2.1%)	n = 207 (12.3%)
**Last Authors**	n = 1413		n = 16 (1.1%)	n = 583 (41.0%)	n = 59 (4.1%)	n = 550 (38.7%)	n = 34 (2.4%)	n = 180 (12.7%)
**Proportion of Countries with Authors**	n = 87/201 (43.3%)		n = 12/47 (25.5%)	n = 16/36 (44.4%)	n = 13/22 (59.1%)	n = 31/55 (56.4%)	n = 2/10 (20.0%)	n = 13/31 (41.9%)

**Table A3:** Full methodology, thematic, and clinical data.

	Total (%)	Global (%)	Africa (%)	Americas (%)	Eastern Med. (%)	Europe (%)	Southeast Asia (%)	Western Pacific (%)
	***Methodologies***							
**Quantitative**	n = 669 (27.7%)	n = 10 (1.5%)	n = 6 (0.9%)	n = 340 (50.8%)	n = 27 (4.0%)	n = 197 (29.4%)	n = 16 (2.4%)	n = 69 (10.3%)
**Qualitative**	n = 561 (23.5%)	n = 16 (2.9%)	n = 16 (2.9%)	n = 217 (38.7%)	n = 24 (4.3%)	n = 208 (37.1%)	n = 15 (2.7%)	n = 71 (12.7%)
**Review/Synthesis**	n = 495 (20.8%)	n = 205 (41.4%)	n = 9 (1.8%)	n = 123 (24.8%)	n = 5 (1.0%)	n = 113 (22.8%)	n = 11 (2.2%)	n = 35 (7.1%)
**Commentary**	n = 477 (20.0%)	n = 32 (5.5%)	n = 6 (1.3%)	n = 226 (47.4%)	n = 11 (2.3%)	n = 163 (34.2%)	n = 8 (1.7%)	n = 33 (6.9%)
**Mixed Methods**	n = 154 (6.5%)	n = 5 (3.2%)	n = 5 (3.3%)	n = 71 (46.4%)	n = 7 (4.5%)	n = 39 (25.3%)	n = 1 (0.6%)	n = 20 (13.0%)
**Protocol**	n = 37 (1.6%)	n = 1 (2.7%)	n = 1 (2.6%)	n = 12 (46.1%)	n = 2 (5.4%)	n = 15 (40.5%)	n = 1 (2.7%)	n = 7 (18.9%)
	***Thematic Areas***							
**Clinicians**	n = 527 (8.12%)	n = 28 (5.3%)	n = 9 (1.7%)	n = 253 (48.0%)	n = 21 (4.0%)	n = 174 (33.0%)	n = 17 (3.2%)	n = 58 (11.0%)
**Parents**	n = 509 (7.8%)	n = 37 (7.3%)	n = 10 (2.0%)	n = 210 (41.3%)	n = 17 (3.3%)	n = 173 (34.0%)	n = 8 (1.6%)	n = 61 (12.0%)
**End of life care**	n = 417 (6.4%)	n = 35 (8.4%)	n = 6 (1.4%)	n = 200 (48.0%)	n = 17 (4.1%)	n = 110 (26.4%)	n = 7 (1.7%)	n = 48 (11.5%)
**Peri-Neonatal Palliative Care**	n = 371 (5.7%)	n = 32 (%)	n = 10 (2.7%)	n = 157 (442.3%)	n = 15 (4.0%)	n = 119 (32.1%)	n = 7 (1.9%)	n = 33 (8.9%)
**Care Models**	n = 285 (4.4%)	n = 28 (%)	n = 1 (0.4%)	n = 108 (38.0%)	n = 8 (2.8%)	n = 109 (38.2%)	n = 7 (2.5%)	n = 24 (8.4%)
**Bereavement**	n = 252 (3.9%)	n = 21 (%)	n = 7 (2.8%)	n = 109 (43.3%)	n = 5 (2.0%)	n = 80 (32.0%)	n = 2 (0.8%)	n = 30 (12.0%)
**Death and dying**	n = 190 (2.9%)	n = 15 (7.9%)	n = 8 (4.2%)	n = 72 (38.0%)	n = 2 (1.1%)	n = 67 (35.3%)	n = 5 (2.6%)	n = 25 (13.2%)
**Decision-Making**	n = 180 (2.8%)	n = 22 (12.2%)	n = 1 (0.6%)	n = 76 (42.2%)	n = 9 (5.0%)	n = 54 (30.0%)	n = 2 (1.1%)	n = 20 (11.1%)
**Needs Assessment**	n = 171 (2.6%)	n = 27 (15.8%)	n = 7 (4.1%)	n = 50 (29.2%)	n = 7 (4.1%)	n = 54 (32.0%)	n = 7 (4.1%)	n = 21 (12.3%)
**Communication**	n = 171 (2.6%)	n = 14 (8.2%)	n = 3 (1.8%)	n = 99 (58.0%)	n = 0 (0.0%)	n = 36 (21.1%)	n = 0 (0.0%)	n = 19 (11.1%)
	***Clinical Focus***							
**Oncology**	n = 447 (41.8%)	n = 38 (8.5%)	n = 13 (2.9%)	n = 224 (50.1%)	n = 18 (4.0%)	n = 90 (20.0%)	n = 15 (3.4%)	n = 53 (12.0%)
**Unspec. Life-limiting/Life-threatening Illness**	n = 298 (27.9%)	n = 42 (14.1%)	n = 3 (1.0%)	n = 110 (37.0%)	n = 6 (2.0%)	n = 103 (34.6%)	n = 3 (1.0%)	n = 32 (10.7%)
**Neurology**	n = 102 (9.5%)	n = 8 (7.8%)	n = 0 (0.0%)	n = 51 (50.0%)	n = 1 (1.0%)	n = 28 (27.5%)	n = 5 (5.0%)	n = 9 (8.8%)
**Cardiology**	n = 55 (5.1%)	n = 4 (7.3%)	n = 0 (0.0%)	n = 43 (78.2%)	n = 0 (0.0%)	n = 6 (11.0%)	n = 0 (0.0%)	n = 2 (3.6%)
**Rare Diseases**	n = 49 (4.6%)	n = 5 (10.2%)	n = 0 (0.0%)	n = 20 (41.0%)	n = 1 (2.0%)	n = 19 (39.0%)	n = 3 (6.1%)	n = 1 (2.0%)

## Appendix B. Full Boolean Operator Search Strategy

(TITLE-ABS-KEY ((“palliative care” OR “hospice care” OR “end of life care” OR “life-limiting condition*” OR “supportive care”) AND (child* OR pediatric* OR paediatric* OR adolesc* OR neonat*)) OR TITLE-ABS-KEY ((“bereavement” OR “grief” OR “mourning” ) AND ( “death of a child” OR “child death”) AND (“parent*” OR “family*” OR “sibling*” )) AND NOT TITLE-ABS-KEY (adult OR geriatric OR aged OR elderly OR dementia)) AND PUBYEAR > 2014.
